# From acute care to chronic disease management: integrating physical exercise into hospital settings

**DOI:** 10.1080/17482631.2026.2719061

**Published:** 2026-09-29

**Authors:** Alejandro Barrera-Garcimartín, Elena Pérez-Calzado, Moisés Marquina, Ignacio Refoyo, Ana López-Martín

**Affiliations:** a Deporte y Entrenamiento Research Group, Departamento de Deportes, Facultad de Ciencias de la Actividad Física y del Deporte (INEF), Universidad Politécnica de Madrid, Madrid, Spain; b Departamento de Ciencias Sociales de la Actividad Física, del Deporte y del Ocio, Facultad de Ciencias de la Actividad Física y del Deporte (INEF), Universidad Politécnica de Madrid, Madrid, Spain; c Oncology Department, Hospital Universitario Severo Ochoa, Leganés, Madrid, Spain; d Facultad de Ciencias Biomédicas y de la Salud, Universidad Alfonso X El Sabio-UAX, Villanueva de la Cañada, Madrid, Spain

**Keywords:** Healthcare systems, physical activity, integrated care, implementation, qualitative, hospital-based care, barriers, facilitators

## Abstract

**Purpose:**

The growing burden of chronic disease, population ageing, and declining physical activity levels are increasing pressure on healthcare systems oriented towards acute care, while exercise remains underused in hospital practice despite its recognised effectiveness and cost-efficiency. This study explored the perspectives of healthcare professionals and exercise specialists on the role of physical activity and exercise in chronic disease care.

**Method:**

A qualitative focus group study was conducted with eight professionals working in a public hospital in Spain, data were examined using inductive thematic analysis.

**Results:**

Participants described patients’ physical activity levels as low. The increasing presence of chronic patients was identified as a challenge for healthcare systems designed to manage acute conditions. Barriers to exercise promotion included limited resources, lack of institutional support and weak regulatory frameworks. Participants highlighted the importance of increasing awareness among patients and healthcare professionals, improving visibility of programmes and fostering collaboration with external institutions. Integrating exercise professionals and developing hospital-based exercise programmes were viewed as important components of a comprehensive approach to chronic disease management.

**Conclusion:**

These findings support a shift toward preventive, long-term, and multidisciplinary care, with stronger collaboration, the integration of exercise professionals and clearer organisational pathways as steps towards more sustainable and patient-centred health systems.

## 1. Introduction

The global transition in lifestyle patterns has been identified as a major driver of contemporary health challenges. Recent evidence indicates that the increasing prevalence of sedentary behaviour, unhealthy dietary practices and chronic stress contributes substantially to the global burden of non-communicable diseases and adverse population health outcomes (Garg, 2025). At the same time, a marked increase in global life expectancy has been observed. However, gains in longevity are not consistently accompanied by corresponding improvements in health status, with many individuals spending a greater proportion of their later years living with disease or functional limitations (Beard et al., 2015).

These factors have contributed to a substantial increase in multimorbidity, such that the typical patient profile has shifted away from acute illness toward the long-term management of multiple chronic conditions (Barnett et al., 2012). The global burden of chronic conditions continues to increase, accounting for nearly three-quarters of all deaths worldwide. Chronic, or non-communicable, diseases refer to a broad group of long-term health conditions that are not transmitted between individuals and typically persist over extended periods. Their development is influenced by a combination of biological predispositions, environmental exposures, and behavioural factors related to lifestyle. Cardiovascular diseases, cancer, chronic respiratory diseases and diabetes represent the most prevalent and impactful categories worldwide (World Health Organisation, 2025). Among non-communicable diseases, cancer represents a major global health challenge. Recent estimates from GLOBOCAN indicate that approximately 20 million new cancer cases and 9.7 million cancer-related deaths occurred worldwide in 2022, with projections suggesting a continued rise in the coming decades (Bray et al., 2024). In Spain, cancer has become the leading cause of death for the second consecutive year, surpassing cardiovascular diseases (INE, 2025).

The growing prevalence of this patient profile, together with increased life expectancy and other structural factors, is contributing to the progressive saturation of healthcare systems across contemporary healthcare settings (Choi et al., 2024). Available evidence suggests that individuals living with chronic conditions make greater use of healthcare resources, including more frequent medical visits and higher rates of hospitalisation compared with those without chronic disease (Polanco et al., 2025). This increased utilisation places additional pressure on healthcare capacity and drives rising healthcare costs. In response, emerging evidence underscores the need to adopt integrated care models that combine primary and secondary prevention, as a means of effectively addressing the expanding burden of chronic disease within contemporary health systems (Murtagh et al., 2021; OECD, 2023).

Physical activity has been widely recognised as a key determinant of health, conferring substantial benefits across a broad range of conditions. Evidence indicates that exercise prescription can be incorporated into the management of up to 26 chronic diseases, and in some cases has been shown to be as effective as pharmacological treatment (Pedersen & Saltin, 2015). Reflecting its relevance, the World Health Organisation (WHO) has developed comprehensive physical activity guidelines, including specific recommendations for individuals living with chronic conditions, recognising physical activity as a safe and beneficial intervention across populations (Bull et al., 2020). However, despite this evidence, translation into hospital-based care remains limited and the levels of physical activity continue to be insufficient. Current estimates indicate that approximately 31% of adults and 80% of adolescents fail to meet recommended physical activity levels. Moreover, ongoing lifestyle changes have been associated with a progressive decline in population-level physical activity (World Health Organisation, 2024). If these trends continue, substantial public health and economic consequences are expected. Projections suggest that by 2030, approximately 499.2 million new cases of preventable non-communicable diseases will occur globally, with the annual economic burden of physical inactivity estimated at around 47.6 billion US dollars (Santos et al., 2023).

In an attempt to address the lack of physical activity in chronic patients during their illness, several countries have included exercise-based programmes in their healthcare systems. Despite increasing recognition of the role of exercise in disease management, the integration of these interventions into routine healthcare remains uneven, in which most are commonly implemented within specific clinical pathways and tend to be delivered during particular stages of care (Dibben et al., 2024). Regulatory and legislative frameworks are still underdeveloped in many countries, resulting in limited formal recognition of exercise professionals within health systems, as exemplified by the situation in Spain. In contrast, some countries have begun to take initial steps toward professional regulation and official recognition. Canada has advanced frameworks for integrating exercise professionals into healthcare pathways (Fortier et al., 2024), and more recently, the United Kingdom has moved toward establishing regulated professional standards and formal recognition within the health system (Jones et al., 2024). The Australian model is widely regarded as the reference standard in this field, where Accredited Exercise Physiologists (AEPs) are formally integrated into the healthcare system. In this context, AEPs specialise in exercise testing and in the prescription and delivery of clinical exercise interventions for individuals with, or at high risk of, chronic and complex medical conditions, injuries and disabilities. Importantly, this model has been shown to be cost-effective, demonstrating both clinical and economic benefits (Deloitte, 2015; Ewald et al., 2018).

There is an urgent need to address effectively the emerging challenges faced by healthcare systems, particularly those related to the growing burden of chronic disease. These changes have prompted the exploration of alternative approaches to complement traditional models of care. Exercise prescription has been shown to be a clinically effective and cost-efficient strategy within this evolving disease paradigm. However, despite the well-established health benefits of physical activity and the generally positive attitudes and awareness among healthcare professionals, exercise prescription is still not routinely implemented in clinical practice (Nauta et al., 2022). This evidence–practice gap appears to be driven by multiple barriers, including resource limitations, time constraints and insufficient professional training (Sun et al., 2024), as well as a lack of consensus regarding the qualifications, roles, functions and responsibilities of exercise professionals within healthcare settings (Shivgulam et al., 2025).

This broader pattern is also clearly reproduced in oncology. Despite robust evidence supporting the benefits of exercise for people living with and beyond cancer, its integration into routine care remains limited. A recent scoping review highlighted that exercise programmes in oncology settings are often fragmented, under-resourced and insufficiently embedded within hospital structures, with a lack of formal policies and clearly defined professional roles (Ezenwankwo et al., 2022) acting as key barriers to implementation. In current clinical practice, exercise-based programmes are typically targeted at specific populations, such as cardiac rehabilitation (Dibben et al., 2023) or cancer prehabilitation (Silver, 2015). This gap highlights the importance of the key agents involved in the prescription, implementation and promotion of exercise as part of chronic disease management namely healthcare professionals and exercise professionals. Although patients play a central role in the management of chronic conditions, the present study deliberately focuses on these professional groups, as previous research has shown their beliefs, knowledge and practices to be critical in facilitating—or hindering—the adoption and maintenance of healthy behaviours, including physical activity, in clinical contexts (Pellerine et al., 2022).

Previous qualitative studies have identified barriers and facilitators affecting exercise counselling, referral and programme implementation in clinical and oncology settings. However, less attention has been paid to how healthcare and exercise professionals with different clinical and organisational roles collectively interpret the position of exercise within hospital care and envisage the pathways required for its integration. This is particularly relevant in healthcare systems such as the Spanish system, where structured exercise services, formal referral pathways and the institutional recognition of exercise professionals remain limited. By bringing together professionals from different disciplines within a shared focus group discussion, the present study extends existing qualitative evidence by examining not only individual perceptions, but also how these perspectives were developed, negotiated, and connected through interaction. In particular, it provides a context-specific and implementation-oriented account of how exercise integration was related to broader questions of chronicity, continuity of care, interprofessional collaboration and coordination between hospital and community-based services.

In view of the situation described, this study aimed to explore how healthcare and exercise professionals connected to a Spanish public hospital understood the current and potential role of physical activity and exercise in the context of chronic disease care, with a particular focus on oncology. The specific objectives were to: (1) examine professionals’ perceptions of patients’ physical activity levels and the factors shaping their engagement in exercise; (2) explore professional and organisational roles, current practices and barriers related to exercise promotion; and (3) examine participants’ views on potential coordinated pathways for integrating exercise across hospital and community settings.

## 2. Materials and methods

This study adopted a qualitative design with an exploratory and interpretative orientation to examine healthcare and exercise professionals’ perspectives on the integration of physical activity and exercise into chronic disease care. This approach was considered appropriate because qualitative enquiry enables the exploration of how individuals and groups understand and assign meaning to a phenomenon within the specific context in which it occurs (Creswell & Poth, 2018).

A focus group methodology was selected because it was considered particularly suitable for bringing healthcare and exercise professionals into dialogue and exploring similarities and differences in their perspectives on the integration of exercise into chronic disease care (Morgan, 1997). This was especially relevant because hospital-based exercise involves professionals occupying different but interdependent clinical, organisational, rehabilitation and exercise-related roles. The interactive discussion enabled participants to compare experiences, respond to and elaborate on one another’s contributions, and express areas of agreement, disagreement, and tension (Kitzinger, 1995). It also allowed perspectives to be collectively developed, qualified, and reconsidered as the discussion progressed (Wilkinson, 1998), providing a contextually situated account of how exercise was understood within a multidisciplinary hospital environment.

The study protocol was approved by the Ethics Committee of Hospital Universitario Severo Ochoa (Nº A1554).

### 2.1. Participants

Participants were purposively selected based on their knowledge and professional experience relating to physical activity, exercise and oncology care. Seven were healthcare professionals employed at Hospital Universitario Severo Ochoa (HUSO; Leganés, Madrid, Spain), while one was an exercise professional with an established collaborative relationship with the institution. This relationship ensured that all participants were familiar with the hospital’s organisational context, clinical pathways and patient population.

The sampling strategy sought variation in professional roles while maintaining a shared oncology-related institutional context. This composition was intended to facilitate the exchange, elaboration, and discussion of perspectives derived from different areas of professional practice. The group was not designed to represent each profession proportionally or to support comparisons between professional categories, but rather to bring together information-rich participants who could contribute different perspectives on the role of exercise in oncology and chronic disease care.

The inclusion criteria were: (I) being aged 18 years or older; (II) having a professional affiliation or an established collaborative relationship with the hospital; (III) having at least six years of professional experience in their respective field (hospital management, oncology, nursing, rehabilitation, physical therapy and training); and (IV) having oncology-related experience, defined as current or previous professional responsibilities involving the care, support, rehabilitation, or exercise guidance of people with cancer.

Participants were recruited between March and May 2025. Eight professionals met the inclusion criteria and agreed to participate. Participation was voluntary, and no financial or other incentives were provided. The three oncology nurses worked in complementary areas of care, including palliative care, outpatient day hospital services and breast cancer management. Participants’ sociodemographic and professional characteristics are presented in Table 1.

**Table 1.** Sociodemographic and professional characteristics of the focus group participants.

Characteristics	Participants (*n* = 8)
Age mean [range]	50 [31−67]
Sex	
Male	2
Female	6
Professional role	
Medical director	1
Oncologist	1
Oncology nurse	3
Rehabilitation physician	1
Physical therapist	1
Exercise Professional	1
Years of professional experience [range]	27 [7−40]
Oncology-related experience [range]	11 [2−25]
Highest educational qualification	
Bachelor’s degree	3
Master’s degree	2
Doctoral degree (PhD)	3

Before the focus group, participants received written information explaining the study objectives, procedures, voluntary nature of participation, and right to withdraw. Written and verbal informed consent were obtained before the session, including explicit permission for audio recording. All procedures were conducted in accordance with the ethical principles of the 2024 revision of the Declaration of Helsinki.

### 2.2. Data generation: focus group discussion

The focus group discussion guide was informed by previous literature in this area (Caperchione et al., 2022; Granger et al., 2016). An initial version was developed by the research team and subsequently reviewed by four experts. Two had expertise in qualitative methodology, particularly in the design and conduct of focus groups, while the other two were professionals with experience directly related to the study topic. Their feedback was used to refine the relevance, clarity, and sequencing of the discussion prompts.

The final guide was organised around two broad areas aligned with the study objectives: the current place of physical activity and exercise within patient care, and the potential development and integration of exercise programmes within the hospital setting. Broad opening prompts were used to introduce each discussion area, while indicative probes were available to clarify participants’ contributions and explore issues arising during the conversation. The guide was used flexibly rather than administered as a fixed sequence of questions. The wording and order of the prompts were adapted to the progression of the discussion, and not all probes were necessarily used. Topics that emerged but fell outside the study objectives, such as issues specifically concerning exercise among women, were acknowledged but not explored in depth (Table 2).

**Table 2.** Focus group discussion guide.

Discussion area	Opening discussion prompt	Indicative probes
Professional context	Please introduce your current professional role and describe your relationship with the hospital and oncology care.	Current responsibilities; involvement in the care, rehabilitation, or exercise guidance of people with cancer.
Current place of physical activity and exercise in patient care	Thinking about the patients you work with, how would you describe the current place of physical activity and exercise in their daily lives and care?	Perceived physical activity levels; factors influencing participation; beliefs about exercise; roles of healthcare and exercise professionals; current health-system initiatives; referral to exercise programmes.
Integration of exercise programmes within the hospital setting	How could physical activity and exercise programmes be incorporated into hospital-based care?	Potential benefits for patients and health services; organisational and professional requirements; resources and institutional support; professional collaboration; referral pathways; implications of developing hospital-based exercise programmes, including programmes for people with lung cancer.

The focus group was conducted on the hospital premises in May 2025. The hospital was selected because it constituted a familiar and accessible setting for participants and represented the institutional context directly addressed in the discussion. This setting was consistent with the study’s aim of generating a contextually situated account of how physical activity and exercise were understood and discussed within this specific multidisciplinary environment (Creswell & Poth, 2018). The session took place in a spacious, well-lit, and temperature-controlled room provided by the hospital. One chair was available for each participant, and the chairs were arranged in a circle so that all participants could see one another and engage directly throughout the discussion. This arrangement was intended to facilitate eye contact, communication, and interaction within the group. Conducting a focus group in a familiar and suitable setting may contribute to creating a comfortable and contextually relevant environment for discussion (Liamputtong, 2011), while providing a clear description of the data collection setting supports transparent reporting in qualitative research (Tong et al., 2007).

The discussion was moderated by a doctoral researcher with experience in qualitative research and in conducting individual interviews and focus groups. Her familiarity with physical activity and exercise facilitated her understanding of profession-specific contributions and the use of relevant follow-up probes. The moderator used participants’ contributions to sustain the discussion and encourage interaction within the group. For example, after one participant raised the importance of developing greater awareness of physical activity, she asked: “Following what has just been said, I would like to ask you what role you think you have, as healthcare or exercise professionals, in promoting physical activity and developing greater awareness around it.” Similarly, when a participant described an initiative implemented in their own professional context, the moderator invited comparison with other settings: “You have done this in your setting, but I would like to know whether similar practices are also being implemented in other contexts, or whether you think current approaches are moving in a different direction”. Participants were also invited to clarify, elaborate on, qualify, or challenge previously expressed perspectives. In this way, the discussion enabled them to compare professional experiences and express areas of both convergence and disagreement (Kitzinger, 1995; Morgan, 1997; Wilkinson, 1998). The moderator sought to balance participation by managing dominant contributions and inviting fewer vocal participants to share their perspectives, while allowing the discussion to develop naturally (Krueger & Casey, 2015). She had no prior personal or professional relationship with any of the participants, in accordance with recommendations for transparent reporting of researcher–participant relationships in qualitative research (Tong et al., 2007).

A single focus group was conducted and lasted 67 minutes. Although the study did not seek to establish saturation across groups, the relevance of the dataset was considered in light of the concept of information power, taking into account the focused study aim, the specificity and professional experience of the participants, the quality of the group dialogue, and the analytical approach (Malterud et al., 2016). The group was therefore considered capable of providing a rich and contextually relevant account of the phenomenon under examination, while recognising that additional groups might have generated further or contrasting perspectives. A comparable single-focus-group design has recently been used to explore the perspectives of experienced multidisciplinary rehabilitation professionals (Christophers et al., 2025).

The focus group was audio-recorded with participants’ consent. No observational field notes were collected during the focus group; consequently, the subsequent examination of group interaction was limited to the verbal exchanges identifiable in the audio recording and transcript.

### 2.3. Data analysis and trustworthiness

After the focus group, the audio recording was transcribed verbatim in Spanish into a Microsoft Word® document. Both researchers listened to the recording and repeatedly read the transcript to become familiar with the data and identify preliminary ideas relevant to the study objectives. The quotations selected for inclusion in the manuscript were subsequently translated into English by the research team.

The analytical team consisted of the principal investigator and the focus group moderator, both of whom had similar professional backgrounds and prior knowledge of physical activity and exercise. The researchers recognised that this familiarity could influence the aspects of the data to which they directed their attention and the interpretations developed during the analysis. A reflexive journal was therefore maintained throughout the analytical process to document preliminary interpretations, decisions, and modifications agreed during research meetings, as well as to reflect on how the researchers’ previous knowledge and expectations might shape the analysis.

An inductive thematic analysis was conducted following the six-phase framework proposed by Braun and Clarke (2006). Coding was conducted primarily at a semantic level, focusing on the explicit meanings expressed by participants. The subsequent development of themes involved a more interpretative examination of broader patterns, relationships, and organisational tensions across the discussion.

Both researchers independently coded the full transcript without using a predefined coding framework. Codes were assigned to segments ranging from individual statements to longer conversational exchanges. Given that the data were generated through a focus group, the analysis considered both the content of participants’ contributions and how perspectives developed through interaction. Particular attention was paid to sequences in which participants supported, elaborated on, qualified, questioned, or challenged one another’s accounts, as well as to areas of convergence and disagreement within the group (Duggleby, 2005; Nicholson & Shrives, 2024; Onwuegbuzie et al., 2009).

Following the independent coding, the researchers met regularly to compare their interpretations and discuss differences in the codes assigned to the data. Through these discussions, codes were clarified, combined, separated, or reformulated, and an agreed coding framework was developed. Related codes were subsequently grouped into themes and subthemes, which were reviewed against the coded extracts and the full transcript. The researchers discussed and refined the themes to ensure that they reflected the main patterns identified in the data while retaining relevant divergent perspectives.

NVivo software (14 version) was used to support the organisation, coding, retrieval, and comparison of the data. Verbatim quotations were selected to illustrate the analytical interpretation of each theme and subtheme. Where relevant, sequential exchanges between participants were retained to demonstrate how perspectives were collectively developed or debated during the focus group. All contributions were anonymised using assigned alphanumeric identifiers (e.g., P1, P2, P3, etc.).

Several strategies were used to support the trustworthiness of the analysis (Lincoln & Guba, 1985). Credibility was supported through repeated engagement with the recording and transcript, independent coding by two researchers, discussion of alternative interpretations, and consideration of both convergent and divergent perspectives. Dependability was supported by documenting analytical decisions and agreed modifications in the reflexive journal. Confirmability was addressed by repeatedly returning to the transcript and coded extracts and by critically reflecting on the researchers’ influence on the analysis. Transferability was facilitated by providing detailed information about the participants, institutional context, focus group setting, data-generation procedures, and scope of the study, enabling readers to assess the relevance of the findings to other contexts.

## 3. Results

Although the discussion was anchored in oncology care, participants did not interpret the challenges surrounding exercise as exclusively cancer-specific. Several participants drew on their broader professional experience, and the interaction progressively shifted from particular care pathways to the broader difficulties hospitals face in responding to patients with long-term and complex needs. Chronicity therefore emerged inductively as an overarching interpretative pattern: not simply as an increase in the number of patients requiring care, but as a mismatch between patients’ long-term needs and a hospital system still organised around acute and time-limited episodes of care. This interpretation was progressively developed through interaction, as participants drew on, extended, and qualified one another’s contributions. A rehabilitation professional initially located this tension within the boundaries of existing service provision:

*“Rehabilitation treatment is not a chronic treatment. In the hospital, we are supposed to treat acute conditions, not chronic ones.”* (P8).

A participant approaching the issue from an organisational perspective then broadened this account from rehabilitation services to the hospital model as a whole:

*“In a way, a new type of patient has emerged, one that did not exist in the hospital before. Twenty years ago, hospitals were made up of acute care units, but there were no chronic patients. You got better, or you did not. But now many conditions have become chronic and continue over time, and hospitals are not prepared for that in terms of resources.”* (P2).

The same participant later intensified this concern by describing chronicity as “a tsunami” that could not be absorbed with the resources currently available. Rather than merely repeating this position, an oncology professional explicitly endorsed it and then qualified its practical implications:

*“I think [P2] is absolutely right. The objective would not simply be for us to organise exercise groups here. Ideally, exercise would be established more broadly as something relevant to every citizen, rather than only to people who are ill. These programmes should act as a bridge to a wider reality in which exercise is integrated across the population.”* (P1).

This exchange reframed an initial concern about rehabilitation provision as a broader organisational challenge, while different professional perspectives highlighted regulatory boundaries, limited resources, growing long-term demand, and the need for continuity across care settings. Chronicity therefore became the shared interpretative lens through which participants discussed both the marginal position of exercise within current care and the development of more coordinated pathways. Accordingly, the findings were organised around two themes: **exercise at the margins of an acute-care-oriented system** and **envisaging a more coordinated place for exercise in chronic care** (Figure 1).

**Figure 1.**
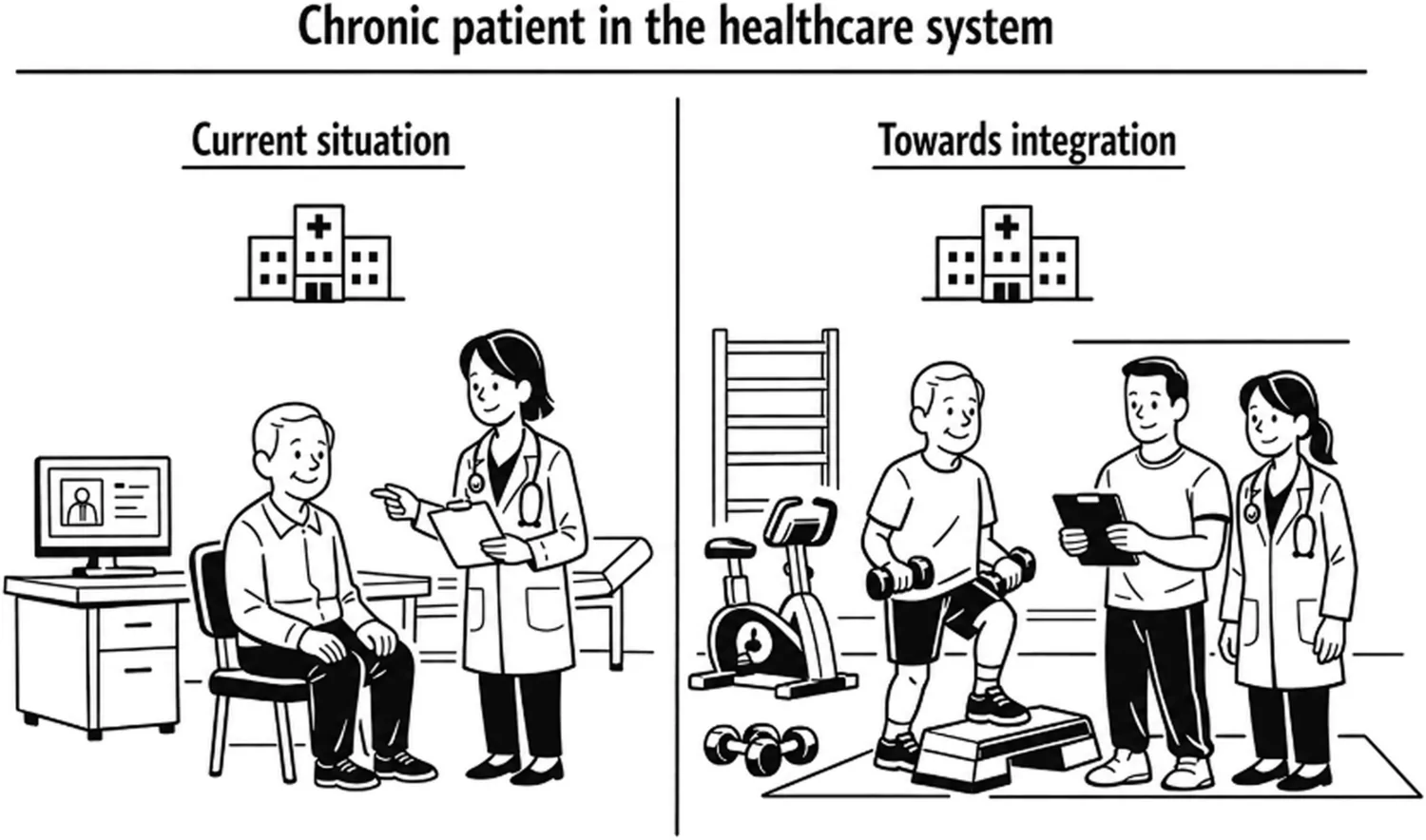
Towards coordinated exercise pathways in chronic disease care: An interpretative summary of participants’ perceptions of current healthcare limitations and proposed directions for exercise integration.

### 3.1. Exercise at the margins of an acute-care-oriented system

#### 3.1.1. Low activity and passive recovery as the prevailing patient profile

Participants initially converged on the view that patients’ physical activity levels were generally low, although they related this pattern to different aspects of their professional experience. The oncologist first linked it to the ageing profile of the population, noting that structured exercise was uncommon and that activity was usually limited to walking. A breast-care nurse extended this observation to younger patients, who also frequently described walking as their only form of exercise, while a palliative-care nurse added that the importance of maintaining muscle mass was often poorly understood. The organisational and exercise perspectives subsequently situated these clinical observations within the wider local context, associating low activity with both the ageing population and the low socioeconomic status of the area.

*What I can share with my colleagues and department heads, when we talk in a more informal way, is that the level of physical activity among the population of Leganés is clearly low.* (P2).

As the discussion progressed, participants moved beyond describing activity levels and interpreted them as reflecting limited awareness of the benefits of physical activity. A nurse argued that exercise was often understood as something undertaken to lose weight or because it had been prescribed, rather than as an habitual means of maintaining health. The oncologist developed this point by explaining that illness frequently intensified inactivity, as patients and their families believed that exercise could be harmful:

*It’s even somewhat misunderstood that they should stop physical activity because it might harm them, when in fact it’s often quite the opposite. That was something that came up frequently. There’s simply no awareness that this not only doesn’t cause harm, but can actually help the progression of the disease, no matter how advanced it may be.* (P1).

Rehabilitation professionals then translated this cultural and illness-related pattern into their own practice. The physiotherapist described patients arriving with the expectation that professionals would move or treat them, rather than actively participating in their recovery. Building on this account, the rehabilitation physician characterised a passive conception of rehabilitation and exercise:

*We often encounter this kind of patient who sees rehabilitation as a completely passive treatment. They don’t view the rehabilitation process, or the recovery process, as something active. Instead, it’s more like, ‘I just come here and I want to be treated or manipulated’.* (P8).

Taken together, the discussion constructed low activity and passive recovery as mutually reinforcing. The ageing profile and low socioeconomic status of the population were perceived as contributing to an already limited exercise culture, while limited awareness of its benefits, the belief that exercise could be harmful, and the experience of illness further encouraged patients and families to withdraw from activity and rely on passive forms of care.

#### 3.1.2. Promoting exercise through awareness and connection to community resources

Participants framed exercise promotion as a process that required more than providing isolated advice. Building on the previous discussion about limited exercise culture, the exercise professional argued that awareness had to be created through a consistent message delivered from the beginning of treatment. The moderator redirected this idea to the group, prompting participants to consider their own role in creating this culture. From physiotherapy and rehabilitation, this role was associated with educating patients about the value of physical exercise and helping them engage in it safely:

*The role of the physiotherapist and our rehabilitation doctors is to start educating people about physical exercise—and about exercising safely, so they don’t get injured.* (P4).

Participants then extended awareness beyond education to include active guidance towards available resources. Rehabilitation professionals described encouraging patients to continue exercising after discharge by directing them to local sports facilities and community programmes. From oncology, referral by a trusted healthcare professional was considered particularly important because it gave patients confidence in the safety of the programme and appeared to support adherence. This position was further developed by the physiotherapy perspective, which constructed the hospital as an initial point of contact rather than the sole provider of long-term exercise:

*We are like the first point of contact in the hospital, so we can act as a filter and guide them. If they enjoy it and get engaged within the hospital setting: ‘okay, this is as far as I can take you, but from here there are many resources available and I can help you access them’. For example, sports centres or the Cancer Association, which also offers programmes.* (P8).

Awareness and continuity were therefore understood as interconnected: professionals should communicate the value of exercise, initiate safe participation, and facilitate connection with community-based resources. Participants also proposed increasing the visibility of existing initiatives through scientific dissemination and social networks. However, this proposal was qualified by concerns that digital communication might not reach many older patients, leading the group to emphasise complementary channels such as health centres, municipalities, older people’s centres, and printed information.

#### 3.1.3. Professional responsibility without organisational support

The discussion about raising patient awareness became increasingly reflexive, as participants questioned whether professionals could promote exercise credibly without first recognising its value themselves. The exercise professional framed professional conviction as a prerequisite for influencing patients, a point subsequently supported by nursing participants:

*Just as we need to make patients aware of how important this is, the first one who needs to be aware is the person who tells them. Otherwise, the message isn’t credible.* (P3).

However, participants also resisted placing responsibility solely on individual professionals. The rehabilitation physician explained that many exercise-related initiatives depended on unpaid work and personal commitment, which could not be expected equally from all staff. This shifted the discussion from professional motivation to the organisational conditions under which such motivation operated.

Resource constraints, service regulations centred on acute care, and the absence of a coordinated national strategy were consequently interpreted as limiting professionals’ capacity to translate awareness into sustainable practice. As the rehabilitation physician noted:

*You have to explain that, unfortunately, it’s not possible nowadays, given that resources are increasingly limited—and we all see this in our consultations. The same thing happens in rehabilitation.* (P8).

Participants therefore constructed a tension between responsibility and capacity: professionals were expected to educate, motivate, and develop initiatives, yet these activities frequently relied on individual effort rather than formal time, resources, or institutional support.

### 3.2. Envisaging a more coordinated place for exercise in chronic care

Building on their shared recognition of the limitations of acute-oriented care, participants envisaged exercise moving from an occasional recommendation towards a more coordinated component of chronic disease management. As one oncology professional stated:

*“We believe that physical exercise should be prescribed as another component of treatment.”* (P1).

As the discussion developed, this position was not equated with hospitals assuming responsibility for all long-term exercise provision. Instead, participants differentiated complementary routes according to patients’ needs and stages of care: referral to trusted community programmes, hospital-based provision for those requiring greater clinical proximity, and collaboration with exercise professionals. Together, these routes were framed as possible ways of improving continuity, safety, and access while responding to existing organisational and resource constraints.

#### 3.2.1. From optional advice to an adjunct component of chronic care

Participants progressively reframed exercise from optional lifestyle advice to a clinically relevant component of chronic care. An oncology professional initially argued that it should be prescribed “as another component of treatment.” The exercise professional extended this position by emphasising its preventive value and favourable cost–benefit balance, linking improved patient health with reduced pressure on services:

*It has an incredible preventive effect that wouldn’t completely, but would significantly, relieve the excessive workload you’re facing now because of the chronic nature of treatments.* (P3).

However, the discussion also indicated that routine prescription could not depend solely on individual professionals or isolated hospital initiatives. From an organisational perspective, local programmes were described as small-scale actions that could increase visibility and gradually support wider change, but participants stressed that their sustainability required coordinated policy and long-term planning. This position was supported from oncology, where the growing recognition of exercise within scientific societies was contrasted with its slower translation into healthcare policy:

*There is no national strategy setting out where we should be heading—in other words, what direction we want to take. We need to take a longer-term view, but that kind of long-range thinking is not exactly common in politics.* (P5).

Exercise was therefore constructed as both a potential adjunct to individual patient care and a broader preventive resource for health services. Nevertheless, participants considered that moving beyond occasional recommendations would require institutional support, coordinated strategies, and evidence capable of demonstrating benefits over the longer term.

#### 3.2.2. Building connected exercise pathways across hospital and community settings

Participants did not frame hospital- and community-based exercise provision as competing alternatives, but as complementary components of a pathway adapted to patients’ clinical needs and stage of care. From an organisational perspective, healthcare professionals could initiate, recommend, and prescribe guided exercise, but its longer-term provision would require collaboration with institutions possessing resources beyond those available within the hospital:

*Medical professionals, nurses, and physiotherapists can recommend and prescribe guided activity, but this also depends on collaboration between institutions.* (P2).

Rehabilitation and oncology professionals subsequently refined this proposal by distinguishing between patients who could transition to community programmes and those who required greater clinical proximity. Hospital-based provision was considered particularly valuable for patients with complex conditions, mobility difficulties, or frequent hospital attendance, as it could provide a safe point of entry into exercise and facilitate timely communication between professionals:

*If I could ask for something, it would be to have an oncology exercise programme here in the hospital, within the hospital setting—especially having all the professionals nearby. For example, if I have a question about a patient, I could ask: ‘Can I work on this?’ or ‘Is the patient too limited?’ There are certain things that might escape me, or training guidelines that other professionals could provide. Something like that, as a team. I think it could work really well and would be great.* (P6).

As patients became more stable and confident, participants envisaged referral to trusted community programmes as a means of sustaining participation beyond hospital care. Direct communication between clinical teams and external providers was considered important for reinforcing patients’ confidence, safety, and adherence. Continuity was therefore understood not as provision in a single setting, but as a connected pathway in which the hospital supported safe initiation and clinically complex cases, while inter-institutional referral enabled longer-term engagement in the community.

#### 3.2.3. Negotiating professional roles within clinically connected exercise pathways

Participants did not frame the inclusion of exercise professionals as replacing healthcare staff, but as distributing responsibilities across complementary areas of expertise. From oncology, exercise professionals were considered able to supervise and progress structured exercise while remaining connected to the clinical team. This coordination was viewed as providing reassurance to patients and enabling situations requiring clinical assessment to be referred back to healthcare professionals:

*They are not healthcare professionals; in this case, they are exercise trainers, but they are trained and guided. They stay in contact with us, which also gives patients a sense of safety, even though there may be specific situations that cannot be managed there and require a healthcare professional to address them.* (P1).

The exercise professional subsequently made this complementarity explicit by rejecting the idea of professional competition and distinguishing between contributions at different stages of care:

*It may sometimes seem that physiotherapists and sport and exercise science professionals are in opposition, but that is not what this is about. Your role is more closely related to the acute phase, while ours is more closely related to the chronic phase. Neither is better than the other; we need to find the best possible ways of collaborating.* (P3).

Exercise provision was therefore constructed as neither an exclusively medical intervention nor an activity disconnected from clinical care. Healthcare professionals retained responsibility for assessment, acute rehabilitation, and the management of clinical risks, while exercise professionals were positioned to tailor, supervise, and progress longer-term exercise. Regular communication between both groups was understood as the mechanism that could allow exercise to remain non-medicalised while still being clinically informed and safe.

## 4. Discussion

The aim of this study was to explore how healthcare and exercise professionals connected to a Spanish public hospital understood the current and potential role of physical activity and exercise in chronic disease care, with a particular focus on oncology. Particular attention was paid to the current hospital context and to the need for innovative strategies capable of addressing ongoing global changes. The results obtained from a focus group involving healthcare and exercise professionals with extensive experience in this field, notably in oncology, provided insights on the realities, challenges and opportunities that characterise the current situation.

Participants identified the consolidation of chronic illness as a disruptive element reshaping contemporary healthcare and exposing a mismatch between patients’ long-term needs and a hospital system predominantly organised around acute and time-limited episodes of care. Within this context, the discussion developed around two interconnected dimensions: the constraints of the existing healthcare system and the directions in which care models might evolve to respond more effectively to this growing challenge. Rather than identifying entirely new barriers to exercise promotion, the contribution of this study lies in showing how professionals with different clinical and organisational roles collectively understood limited resources, unclear professional responsibilities, weak institutional support, and discontinuity between hospital and community services as interconnected rather than isolated challenges.

Contemporary societies are undergoing continuous transformation, characterised by changes in lifestyles, technological development and cultural patterns that have progressively shaped population health (Gulis et al., 2025). Advances in medicine and public health have led to substantial increases in life expectancy, largely due to the effective control of acute and infectious diseases. As a result, patterns of morbidity and mortality have shifted, with chronic, non-communicable conditions becoming the predominant health challenge worldwide (World Health Organisation, 2025). Within this epidemiological transition has contributed to the emergence and consolidation of the chronic patient as a defining feature of modern health systems, with an increasing number of individuals living longer with long-term conditions (Bhuiyan et al., 2024; Thomas et al., 2023).

Societal changes have contributed not only to increased life expectancy but also to fundamental shifts in lifestyle patterns. Participants in the focus group emphasised the progressive ageing of the population, and that older individuals often exhibit low levels of physical activity, a combination that substantially increases the risk of chronic disease development and progression. This combination has important implications for health, as physical inactivity among older adults has been consistently associated with a higher prevalence of chronic conditions and poorer clinical outcomes (DiPietro, 2001). Physical inactivity has been increasingly described as a major global public health challenge, to the extent that it has been referred to as a “new pandemic” (Kohl et al., 2012). In line with this perspective, the participants in the present study reported that, beyond generally low levels of physical activity, walking was often the predominant or sole form of activity performed by most patients. This pattern is consistent with existing literature, which suggests that walking is commonly reported as the most frequently adopted form of physical activity in the general population and, in many contexts, the default option when people report being “active” (Guglielmo et al., 2021; Lesser et al., 2021). However, this is particularly concerning, as walking alone —especially when undertaken at low intensity or in insufficient volume— is unlikely to meet current international physical activity recommendations, which emphasise achieving adequate weekly amounts of moderate-to-vigorous activity (Bull et al., 2020) while also providing an insufficient stimulus for adaptation. Consequently, according to WHO guidelines, most patients would therefore fail to achieve the minimum levels of moderate to vigorous physical activity and muscle-strengthening exercise required to obtain health benefits.

In addition, participants highlighted the potential influence of patients’ socioeconomic status on these factors. Lower socioeconomic status has been associated with reduced health literacy and lower levels of awareness regarding health-related behaviours, which may negatively affect patients’ ability to engage in preventive and health-promoting practices (Stormacq et al., 2019). This lack of awareness may become even more pronounced following the onset of illness. In the context of cancer, the period surrounding diagnosis represents a particularly vulnerable phase, during which patients frequently reduce their physical activity levels (Fram et al., 2024). Within this context, participants emphasised the critical role of healthcare professionals in patient education, noting that medical advice and counselling on physical activity substantially increase the likelihood that patients adhere to WHO physical activity guidelines (Kettle et al., 2022; Orrow et al., 2012). Beyond individual counselling, greater visibility of existing physical activity and exercise programmes was identified as a key requirement, both within healthcare settings and through broader scientific dissemination strategies. Furthermore, the potential of social media platforms was highlighted as a tool to enhance patient awareness and engagement, given their growing influence in contemporary health communication (Moorhead et al., 2013).

However, awareness should not be directed exclusively towards patients. Participants also stressed the importance of fostering education and awareness among healthcare professionals, noting that it is difficult to promote physical activity if clinicians themselves lack knowledge or confidence in the message they convey. Previous studies have reported limited training on physical activity within some medical school curricula (Weiler et al., 2012). Although most healthcare professionals acknowledge the importance of exercise, relatively few routinely prescribe or recommend it to patients (Baillot et al., 2018; Nauta et al., 2022). Consistent with this, a Canadian study found that only 9.6% of patients had at least one reference to exercise documented in their electronic medical records (Lindeman et al., 2022). These findings highlight the need to prioritise awareness and education initiatives among healthcare professionals as a prerequisite for effectively promoting physical activity among patients.

Participants also highlighted a persistent lack of resources, together with insufficient legislation and regulatory frameworks to adequately address this challenge. From an implementation science perspective, these findings illustrate how the integration of evidence-based practices may be influenced by interacting determinants operating at professional, organisational and wider system levels (Damschroder et al., 2022). In this case, regulatory gaps and the absence of coordinated policy frameworks intersected with limited institutional resources and support. As a consequence, many existing initiatives rely largely on individual professional commitment rather than on formal organisational structures. This dependence may compromise their continuity over time, increase professionals’ workload, and make programmes particularly vulnerable to changes in individual availability and motivation. In addition, current regulations continue to conceptualise healthcare settings primarily as spaces for the management of acute conditions, offering limited responses to the growing burden of chronic disease (García-Goñi et al., 2012; Holman, 2020). These findings suggest that promoting exercise within routine care requires not only motivated and adequately prepared professionals, but also supportive organisational structures, clearly defined responsibilities, sufficient resources, and coordinated regulatory and policy frameworks. Such changes may help align healthcare provision more closely with the evolving social and health-related needs of contemporary populations.

The literature continues to emphasise the need to develop health systems capable of providing long-term care (Beard et al., 2015), as chronic care demands now substantially outweigh acute care needs at a global level (Zuniga et al., 2025). Against this backdrop, participants identified physical activity as a particularly relevant and necessary tool to address these emerging health demands. Physical inactivity has been consistently associated with the development and progression of chronic diseases, underscoring the role of physical activity as a key component of both primary and secondary prevention strategies (Durstine et al., 2013). In addition, participants highlighted the favourable cost–benefit profile of physical activity interventions, a finding that aligns with evidence previously reported in the literature (Deloitte, 2015; Ewald et al., 2018; Guillon et al., 2018; Jorge et al., 2022).

### 4.1. Developing coordinated pathways for exercise in chronic disease care

Given the limitations of current Spanish health system in adequately meeting the needs of people with chronic conditions—in a context of rising demand and finite healthcare resources—this article argues for a transition towards a new paradigm in which physical exercise is more systematically integrated as an adjunct care strategy. From this perspective, implementing different exercise-based routes (e.g., exercise prescription, referral to structured programmes, and/or hospital-based provision when needed) could help partially relieve pressure on services by strengthening prevention, supporting self-management and improving continuity of care. Ultimately, this approach has the potential to enhance health and quality of life among people with chronic disease, particularly in oncology.

As discussed throughout the article, the prescription of exercise within healthcare settings remains low, despite widespread awareness of its potential benefits for patients. This gap has been attributed to multiple barriers, including limited time, insufficient specific training, lack of resources and inadequate institutional support (Jakubiak et al., 2025). In response to these constraints, professionals highlighted the importance of collaborating with reliable external institutions to which patients could be referred. Although some effective programmes and examples of good practice were identified, these collaborations were perceived as insufficient, highlighting the need for greater structural support and the development of coordinated national strategies to address physical inactivity as a major public health challenge (World Health Organisation, 2018).

Several participants highlighted the potential value of integrating exercise professionals within clinical settings. According to their views, such integration could be particularly beneficial for two main reasons. First, some patients may experience difficulties accessing external facilities where exercise programmes are delivered, underscoring the need for hospital-based, on-site exercise interventions. Second, the inclusion of these professionals could contribute to the development of more interdisciplinary teams within hospitals, bringing together expertise from different fields and enabling a more holistic approach to the management of chronic and complex conditions (Fairag et al., 2024; Frajacomo & Deminice, 2023). Importantly, the incorporation of exercise professionals into healthcare settings is not a novel concept, but rather a long-standing recommendation within the literature (Cormie et al., 2018; Franklin et al., 2009). Their integration has been suggested as a way to facilitate the shift from predominantly acute-focused healthcare models towards approaches better suited to the long-term management of chronic disease through the inclusion of exercise professionals and structured exercise programmes (Dalzell et al., 2017).

### 4.2. Limitations and future lines

This study has several limitations that should be considered when interpreting the findings. First, the focus group included professionals working within a relatively specific clinical area, namely oncology, which may have shaped the perspectives expressed and limits the applicability of the results to other clinical populations. Although chronicity emerged as a central pattern, the study was primarily situated within an oncology-related context. Therefore, the relevance of these findings to other chronic conditions and clinical pathways should be examined in studies involving a broader range of specialties and healthcare settings. Participants were also drawn from a single public hospital, and only one focus group was conducted. Additional focus groups involving professionals from other hospitals, healthcare systems, and clinical contexts could have generated further or contrasting perspectives and enabled comparison across different organisational cultures and care pathways.

The sample consisted of professionals with a particular interest in or experience of physical activity and exercise, which may have introduced selection bias and resulted in more favourable perspectives towards exercise promotion than might be observed among the broader healthcare workforce. Finally, the study focused specifically on physical activity and exercise without considering other complementary strategies that may also contribute to the comprehensive management of chronic disease, such as nutrition.

Future research should therefore explore these issues across different hospitals, clinical areas and institutional contexts. Including a wider range of healthcare and exercise professionals, hospital managers, policy-makers, patients, and other stakeholders involved in organisational and regulatory decision-making could provide a more comprehensive understanding of current practices, implementation barriers and the conditions required to integrate exercise into chronic disease care.

The conceptual directions identified in this study should not be interpreted as establishing a new paradigm. Rather, they represent a preliminary and context-specific account of how a multidisciplinary group of professionals envisaged a healthcare model in which exercise could assume a more prominent role in chronic care. Further research across diverse populations and settings is needed to examine the extent to which these perspectives are shared, to identify alternative or conflicting views, and to determine whether they may contribute over time to the development of a broader conceptual approach to the integration of exercise within healthcare.

## 5. Perspectives for implementation

As highlighted throughout this article, healthcare and exercise professionals consistently perceived exercise as a highly valuable component of care, with potential benefits extending across multiple dimensions of chronic disease management. Many professionals reported attempting to promote or incorporate exercise within their routine practice. However, these efforts continue to be constrained by major structural barriers, particularly the lack of resources, formal support and coordinated national strategies to guide implementation.

In this context, the present study seeks to contribute to an ongoing call for action, in line with initiatives already emerging in countries such as the United Kingdom (Jones et al., 2024) and Canada (Fortier et al., 2024), and to encourage progress toward models that are currently regarded as benchmarks, such as Australia. Advancing in this direction will require the development of networks, institutional structures and public policies capable of supporting professionals and facilitating clear referral pathways for exercise-based care.

A key priority identified by participants was the need to improve awareness and training among healthcare professionals, who remain central to the delivery of care and to the promotion of healthy behaviours. Without greater professional engagement and understanding, exercise is unlikely to become fully embedded within routine practice. At the same time, participants stressed that these initiatives cannot continue to depend solely on individual motivation or personal time. Instead, they must be supported by formal structures, policies and organisational frameworks that enable their sustainable implementation.

Once such awareness and structural support are in place, more effective systems of referral and exercise prescription could be developed, allowing chronic disease to be addressed through a more holistic and multidisciplinary approach. Within this model, the inclusion of exercise professionals may play an important role, whether through partnerships with external organisations or through their direct integration into hospital settings, where they could contribute to the design and delivery of tailored exercise programmes.

Taken together, these lines of action may help relieve the growing pressure placed on healthcare systems by the increasing number of patients living with chronic conditions. Their relevance and potential impact, however, are likely to depend on the organisational, regulatory, and resource characteristics of each healthcare setting, and their transferability should therefore be considered in relation to the similarity of other contexts to that examined in this study. Where appropriately adapted and supported, moving in this direction could contribute to improved health outcomes, reduced healthcare expenditure, and enhanced quality of life for patients. The potential benefits are already clear, the pressing question is no longer whether action is needed, but how much longer health systems can afford to delay it.

## 6. Conclusion

This study provides relevant information on the perspectives of healthcare and exercise professionals regarding current physical activity and exercise role in the context of chronic disease care, with a particular focus on oncology. Overall, participants consistently reported low levels of physical activity among patients, with walking identified as the most common—and often the only—form of activity undertaken. These findings were discussed in the context of a growing patient profile that was not originally envisaged within healthcare systems predominantly designed to manage acute conditions: the chronic patient.

To address this challenge, participants described a range of strategies currently employed to encourage physical activity, including increasing the visibility of existing programmes and enhancing patient awareness. However, they also highlighted the limitations of these approaches in the absence of sufficient resources, coordinated national strategies and greater awareness among some healthcare professionals.

In this context, exercise programmes were identified as a promising component of comprehensive care for patients with chronic conditions, with potential benefits for both primary and secondary prevention. Participants emphasised the importance of intersectoral collaboration, particularly with external institutions, to support programme implementation and patient referral pathways, as well as the potential value of integrating exercise professionals within hospital environments.

Taken together, these findings support the need to move towards a model of chronic disease care in which exercise is fully integrated as a core component of prevention, treatment and long-term management. In the context of population ageing, rising multimorbidity and growing pressure on healthcare systems, such a shift may be essential not only to improve health outcomes and quality of life, but also to safeguard the long-term sustainability of care.

## Data Availability

The materials and data generated during this study are available from the corresponding author on request.
